# A Curious Case of Right Upper Quadrant Abdominal Pain

**DOI:** 10.5811/westjem.2016.7.31011

**Published:** 2016-08-08

**Authors:** Andrew Grock, Wendy Chan, Ian S. deSouza

**Affiliations:** * Olive View, UCLA Medical Center, Department of Emergency Medicine, Sylmar, California; † SUNY Downstate Medical Center, Department of Emergency Medicine, Brooklyn, New York; ‡ Kings County Hospital Department of Emergency Medicine, Brooklyn, New York; § University of Southern California Medical Center and Keck School of Medicine, Department of Emergency Medicine, Los Angeles, California

## Abstract

An otherwise healthy 36-year-old man presented with sudden-onset right upper quadrant abdominal pain and vomiting. A bedside ultrasound, performed to evaluate hepatobiliary pathology, revealed a normal gallbladder but free intraperitoneal fluid. After an expedited CT and emergent explorative laparotomy, the patient was diagnosed with a small bowel obstruction with ischemia secondary to midgut volvulus. Though midgut volvulus is rare in adults, delays in definitive diagnosis and management can result in bowel necrosis. Importantly, an emergency physician must be able to recognize bedside ultrasound findings associated with acutely dangerous intrabdominal pathology.

## INTRODUCTION

Fifty percent of midgut volvulus cases present within the first month of life, and 90% in the first year.^1^ After three months, malrotation with midgut volvulus is considered rare.^2^ Midgut volvulus begins with incomplete embryologic midgut rotation or fixation. Later the bowel can twist, leading to obstruction of the bowel and its mesenteric blood supply.^3^ Typically, midgut volvulus presents with sudden onset abdominal pain with bilious vomiting in the neonate.^4^ It can progress to peritonitis, bowel necrosis, shock, and death.^3^ In this adult patient, a similar presentation initially led to a work-up for gallbladder pathology, which revealed unexpected red flags for dangerous pathology and led to expedited care. A delay in diagnosis may have ultimately resulted in bowel infarction and death.

## CASE REPORT

**Figure 1:**
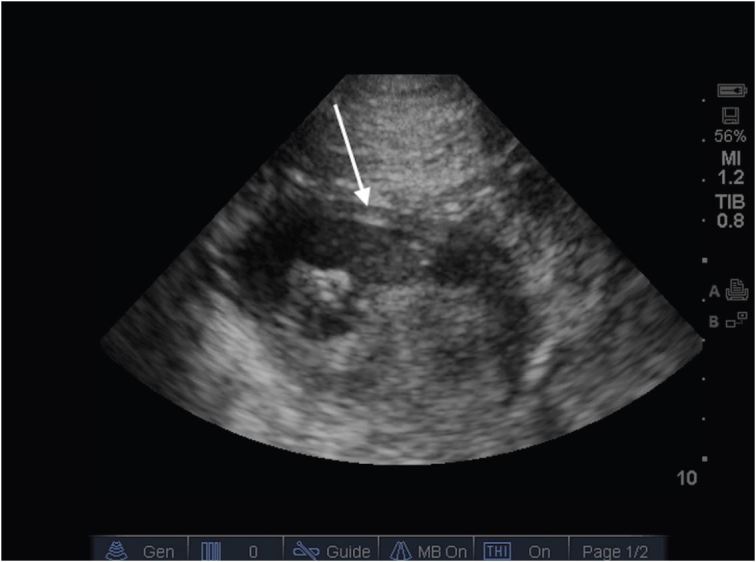
Bowel in transverse view demonstrating distention and wall edema

A 36 year-old male with no past medical history called emergency medical services after he developed acute-onset, constant, right upper quadrant pain and one episode of non-bloody, non-bilious vomiting. Upon arrival to the emergency department (ED), his pain was rated 9/10 and his vital signs were temperature 97 F (36.1 C), pulse 61, respirations 18, blood pressure 155/103, and O_2_ saturation 100%. His examination revealed a firm abdomen, significant right upper quadrant tenderness, and voluntary guarding. Soon after the initial exam, the patient had a second episode of non-bloody, non-bilious vomiting for which he was given ondansetron, ranitidine, and one liter of 0.9% normal saline. A prompt point-of-care ultrasound (POCUS) demonstrated a normal gallbladder. However, it also showed free intraperitoneal fluid in Morison’s pouch and a loop of distended bowel with wall edema (Figure 1). An upright chest radiograph did not demonstrate pneumoperitoneum or any other abnormality. During this period of investigation, the patient’s pain increased in intensity, he exhibited multiple episodes of vomiting, and his abdomen became more rigid.

Presuming an acute abdomen, we contacted surgery for emergent consultation and transported the patient for an expedited computed tomography (CT) abdomen and pelvis with intravenous contrast. The venous lactate level returned at 6.1 mmol/L which prompted the administration of a bolus of two additional liters of normal saline using pressure bags.

The attending radiologist interpreted the CT:

“Malrotation with the third portion the duodenum...Multiple dilated loops of jejunum with suggestion of wall thickening…Distorted mesenteric anatomy with a swirled appearance of the mesenteric vessels. Given this constellation of findings, the appearance is suspicious for a volvulus with early or partial bowel obstruction.”

The patient was taken emergently to the operating room where midgut volvulus was confirmed. According to the perioperative documentation, the entire small bowel was black, and upon manual detorsion, portions of the bowel regained its normal, pink color indicating restored perfusion. The assisting pediatric surgeon successfully performed a Ladd Procedure, and the abdomen was left open.

On the second hospital day, another evaluation in the operating room revealed bowel with both venous congestion and edema but without necrosis. The bowel appeared both viable and peristaltic. On the fourth hospital day, a third and final inspection demonstrated pink and healthy bowel with decreased edema. After decompressing the gastrointestinal tract, the abdomen was closed. The patient was discharged on his ninth hospital day without any additional complications and has continued to do well during follow-up visits.

## DISCUSSION

Malrotation is defined as an abnormal twisting of the small intestines during embryological development, whereas volvulus refers to the condition when this abnormal twisting results in bowel and vascular obstruction.^3^ Midgut volvulus overwhelmingly presents in the first month of life and rarely in adults.^2^ Aside from the issue of age, the diagnosis is complicated in adults as clinical features of midgut malrotation with or without volvulus significantly differ from those in neonates. Adults may present with intestinal obstruction, chronic abdominal pain, malabsorption and diarrhea, peritonitis and septic shock, solid food intolerance, common bile duct obstruction, and abdominal distension.^5^ A common presenting symptom such as vomiting may be absent in 73% of adults. Whereas, in pediatric cases it is absent in only 7%.^4^ As a result, delays in definitive diagnosis of adults with midgut malrotation are common.^6^ In 32% of adult patients, many years may pass before the diagnosis of malrotation is confirmed with the appropriate diagnostic test.^4^,^7^ This delay should be concerning given that malrotation has been called a “time bomb” that can lead to “devastating intestinal necrosis” once volvulus occurs.^4^ Ischemia and pressure necrosis can rapidly lead to intestinal perforation in as few as eight hours, truly making this a surgical emergency.^8^,^9^

CT findings for midgut volvulus fall into three categories: Mesenteric ischemia, small bowel obstruction, and anatomic abnormalities. A finding specific to midgut malrotation is the “whirlpool sign,” which refers to the swirled appearance of the mesentery and superior mesenteric vein (SMV) when they are wrapped around the superior mesenteric artery (SMA) (Image 2).^10^,^11^ Ultrasonography (US) can identify volvulus as well. The two main US findings associated with midgut malrotation are transposition of the SMA and the SMV (sensitivity 67%–100%, specificity 75%–83%) and the whirlpool sign (sensitivity 81–92%, specificity 92–100%).^12^–^15^ In SMA and SMV transposition, the SMV is located on ventral left of the SMA; however, this finding is not always seen in malrotation, and even when present, the patient may be asymptomatic.^12^ The two vessels are identified by using color Doppler to distinguish arterial from venous flow.^13^ Traditionally, the diagnostic gold standard for malrotation is an upper gastrointestinal series (sensitivity of 54–79%), but its low yield and invasiveness negate its practicality in the ED.^15^,^16^

Emergency physicians are focused on differentiating between sick and not sick. In this patient, the POCUS findings of intraperitoneal free fluid and bowel wall edema significantly expedited specialty consultation and confirmatory diagnostic testing. Without POCUS, manual detorsion in the OR would have been delayed, and the marginally viable bowel may not have been saved. The use of early ultrasonography is well supported by the literature. In two recent studies, POCUS helped the provider in 83–87% of patients with undifferentiated acute abdominal pain.^18^,^19^ Importantly, it changed clinical decision-making in 22–47% of patients; this included preventing intended laparotomies or revealing a diagnosis other than the initial clinical impression.^18^,^19^ In other instances, POCUS can obviate the need for further imaging such as in suspected renal and biliary colic.^19^,^20^ The use of POCUS to allow reduced utilization of computed tomography (CT) may have a significant public health benefit, with an estimated 1.5–2.0% of all cancers in the United States attributable to radiation from CT.^23^,^24^

In this case, the finding of intraperitoneal free fluid on POCUS raised the provider’s level of suspicion for acutely dangerous pathology. In a retrospective review of pediatric patients with acute abdominal pain, free fluid on diagnostic imaging doubled the rate of a surgical condition from 25% to 57.4% (p<0.001) as well as increased the rate of surgery from 6.3% to 94.4% (p<0.001).^24^ While free fluid alone is not specific for a surgical pathology, providers must consider this finding when deciding to obtain or prioritize specialty consults and more specific imaging such as CT in these patients. Additionally, the POCUS in this case identified dilated bowel with wall edema. Ultrasonography has also been shown to be useful in identification of bowel obstructions and may serve as the sole imaging modality.^25^ Using the diagnostic criteria of >2.5 cm dilated loops of bowel proximal to collapsed loops of bowel and absent or decreased peristalsis activity, POCUS had a +LR of 9.55 (95% CI = [2.16 to 42.21]) and − LR 0.04 (95% CI = [0.1 to 0.13]) for intestinal obstruction.^25^ In the appropriate clinical setting, such findings on POCUS should increase the provider’s suspicion for intestinal obstruction and need for prioritization of that patient’s care.

A patient with right upper quadrant pain and vomiting may reasonably be suspected to have gallbladder disease. POCUS in this case did not reveal gallbladder pathology but did serve to identify an extremely sick patient with an alternative diagnosis and resulted in an acceleration and prioritization of this patient’s care. Delays in care could have been catastrophic for this patient. While midgut volvulus case reports and series have been published in the surgical literature, this diagnosis has had limited representation in the emergency medicine literature. Emergency physicians should be aware of this diagnosis as well as the sonographic findings in the abdomen that are predictive of pathology that may require surgical intervention. Care of these patients must be expedited, as a delay to definitive diagnosis and treatment may lead to increased morbidity and mortality. In patients with midgut volvulus with associated small bowel obstruction and ischemia, “time is bowel.” An awareness of this potentially life-threatening diagnosis can reduce time to confirmatory testing and definitive treatment in the operating room.

## Figures and Tables

**Figure 2:**
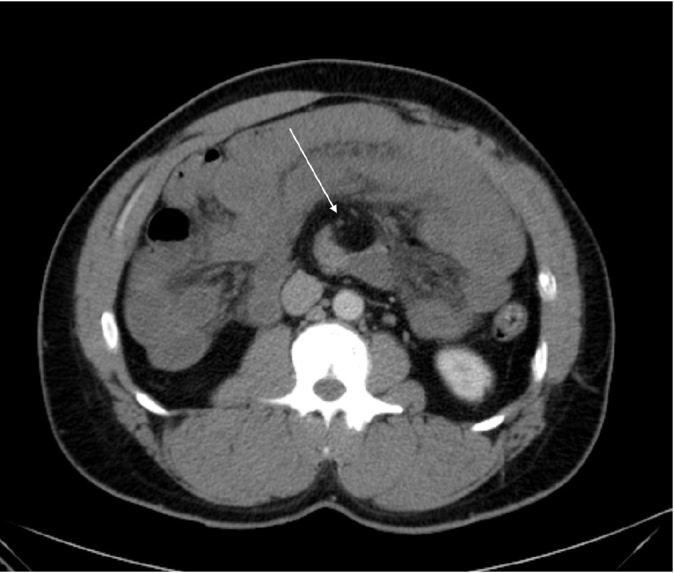
Arrow indicates whirlpool sign
